# Isolation and structural comparison of Ru^II^-dnp complexes [dnp = 2,6-bis­(1,8-naphthyridin-2-yl)pyridine] with axially or equatorially coordinating NCS ligands

**DOI:** 10.1107/S2056989022004443

**Published:** 2022-05-06

**Authors:** Tsugiko Takase, Takashi Yamanaka, Chihiro Tamura, Dai Oyama

**Affiliations:** a Department of Natural Sciences and Informatics, Fukushima University, 1, Kanayagawa, Fukushima 960-1296, Japan; b Graduate School of Science and Engineering, Fukushima University, 1 Kanayagawa, Fukushima 960-1296, Japan; Vienna University of Technology, Austria

**Keywords:** crystal structure, ruthenium complex, polypyridine, tridentate ligand, thio­cyanato ligand

## Abstract

The mol­ecular and crystal structures of two ruthenium(II) complexes, *viz. cis*-aqua­[2,6-bis­(1,8-naphthyridin-2-yl)pyridine-κ^3^ *N*,*N*′,*N*′′](thio­cyanato-κ*N*)(tri­phen­yl­phosphine-κ*P*)ruthenium(II) hexa­fluorido­phosphate–acetone–water (1/0.5/1), [Ru(NCS)(C_21_H_13_N_5_)(C_18_H_15_P)(H_2_O)]PF_6_·0.5C_3_H_6_O·H_2_O (I) and *trans*-[2,6-bis­(1,8-naphthyridin-2-yl)pyridine-κ^3^’*N*,*N*′,*N*′′]bis­(pyridine-κ*N*)(thiocyanato-κ*N*)ruthenium(II) thio­cyanate, [Ru(NCS)(C_21_H_13_N_5_)(C_5_H_5_N)_2_]NCS (II), with an N-coordinating thio­cyanato group and a tridentate polypyridyl supporting ligand, are reported. The Ru^II^ atom in each of the cationic complexes adopts a distorted octa­hedral coordination sphere, defined by an N atom of the thio­cyanato ligand, three N atoms from the tridentate polypyridyl ligand, and an O and P atom in (I) or two pyridine-N atoms in (II) derived from monodentate ligands. The thio­cyanato ligand in (I) coordinates in an axial manner to the {Ru-dnp} unit [dnp = 2,6-bis­(1,8-naphthyridin-2-yl)pyridine], whereas it coordinates in an equatorial manner in (II). In the crystal structure of compound (I), intra­molecular C—H⋯O, C—H⋯N and O—H⋯N hydrogen bonds as well as π–π contacts are present, in addition to inter­molecular C—H⋯F, C—H⋯O and O—H⋯O hydrogen bonds. In the crystal structure of compound (II), intra­molecular C—H⋯N hydrogen bonds are observed along with inter­molecular C—H⋯N and C—H⋯S hydrogen bonds as well as a π–π inter­action.

## Abstract 2

The crystal structures of two Ru^II^ complexes bearing a tridentate polypyridine ligand and N-coordinating thio­cyanato ligands at the axial or equatorial position are compared.

## 1. Chemical context

Polypyridyl­ruthenium(II) complexes play essential roles in key technologies, such as solar energy conversion (Lewis, 2007 ▸). In particular, Ru^II^ complexes with thio­cyanate ion(s) are inter­esting as dye mol­ecules for dye-sensitized solar cells (Hagfeldt *et al.*, 2010 ▸). As a ligand, the thio­cyanate group can bond to metals through the terminal nitro­gen or sulfur atoms since it is ambidentate. Linkage isomeric pairs can be distinguished using spectroscopic techniques when they exist as a mixture (Brewster *et al.*, 2011 ▸; Vandenburgh *et al.*, 2008 ▸). However, identifying the coordinating atom (N or S) by structural analysis is more reliable when only one isomer exists.

A series of Ru^II^ complexes containing a supporting ligand, dnp [dnp = 2,6-bis­(1,8-naphthyridin-2-yl)pyridine], were synthesized to extend the π-conjugated system of the terpyridine framework (which is a typical tridentate polypyridyl ligand) and their properties and reactivities reported (Oyama *et al.*, 2013 ▸, 2017 ▸). In particular, some reactivities such as ligand substitutions are significantly different in an identical coordination framework when the axial ligands are tri­phenyl­phosphine (PPh_3_) or pyridine (py) (Oyama *et al.*, 2013 ▸, 2017 ▸). 
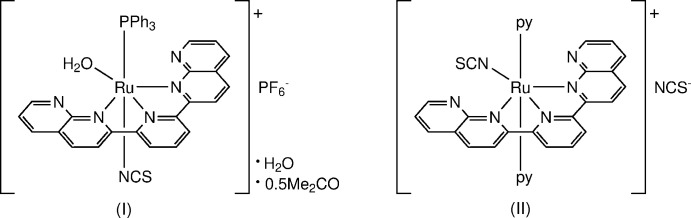


During the current study, the reaction of precursors with different axially bound ligands with the NCS^−^ ion resulted in the formation of the cationic complexes *cis*(PPh_3_,H_2_O)[Ru(dnp)(PPh_3_)(NCS-κ*N*)(H_2_O)^+^ [(I) as the water/acetone (1/0.5) solvated PF_6_^−^ salt] with an axially bound NCS^−^ ligand and *trans*(py)-[Ru(dnp)(py)_2_(NCS-κ*N*)]^+^ [(II) as the NCS^−^ salt] with an equatorially bound NCS^−^ ligand. Their crystal structures are reported and compared in this communication.

## 2. Structural commentary

**Figure 1:**
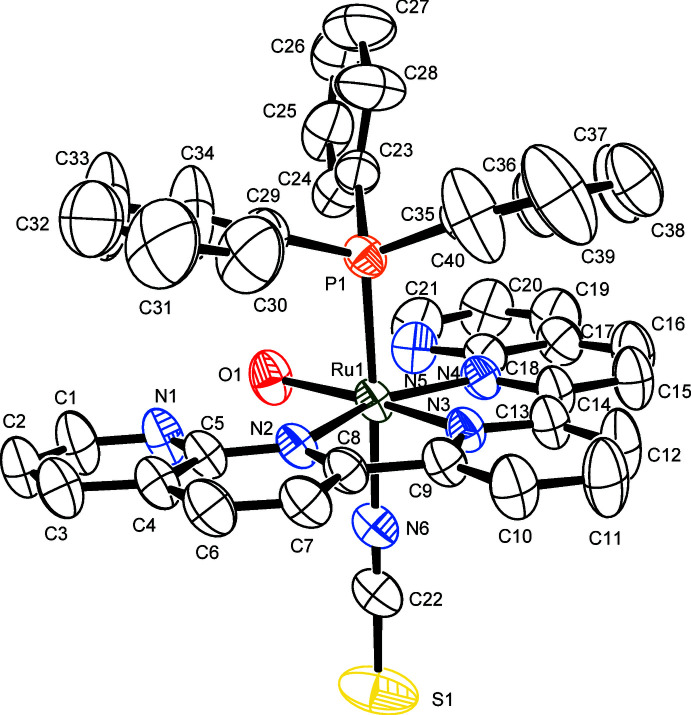
Mol­ecular structure of the complex cation in (I), with atom labels and displacement ellipsoids for non-H atoms drawn at the 50% probability level.

Figs. 1 ▸ and 2 ▸ present the mol­ecular structures of compounds (I) and (II), respectively. The Ru^II^ atoms in (I) and (II) exhibit distorted octa­hedral coordination environments, similar to those reported in other structurally related complexes containing the tridentate dnp ligand (Koizumi & Tanaka, 2005 ▸; Oyama *et al.*, 2013 ▸, 2017 ▸). As listed in Tables 1 ▸ and 2 ▸, compounds (I) and (II) exhibit intra­molecular hydrogen bonds between aromatic C—H groups of PPh_3_ or pyridine and the non-coordinating N atoms in dnp or the monodentate ligands [*O*H_2_ in (I) or *N*CS in (II)]. In (I), the inter­atomic distances between O1 and N1 [2.678 (4) Å] and O1 and N5 [2.983 (4) Å] are considerably short. Although the H atoms of the coordinating water mol­ecule (O1) have not been localized, these short distances indicate that intra­molecular hydrogen bonds of medium strength are present between the aqua ligand and the N atoms of the dnp ligand. Furthermore, in (I) intra­molecular π–π inter­actions [*Cg*1⋯*Cg*2 = 3.640 (4) Å and *Cg*3⋯*Cg*4 = 3.749 (3) Å where *Cg*1, *Cg*2, *Cg*3, and *Cg*4 are the centroids of the N1/C1–C5, C29–C34, N3/C9–C13, and C35–C40 rings, respectively] are present, with a slippage of 1.2 Å for *Cg*1⋯*Cg*2. It is inferred from these results that both π–π inter­actions are not exactly cofacial. The slippage angle *β* is 19.2° for *Cg*1⋯*Cg*2 and 16.2° for *Cg*3⋯*Cg*4.

As mentioned above, it is important to distinguish the coordination atom of the thio­cyanato ligand because of its ambidentate coordination mode. Both S- and N-coordinated Ru^II^ complexes containing polypyridines have been determined structurally, but the N-atom coordination is overwhelmingly dominant. These complexes can be distinguished crystallographically by the Ru—*X*—C bond angle (*X* = N or S) through the coordinating atom. For example, the Ru—S—C bond angles (for S-ligating examples) are 104–106° (Brewster *et al.*, 2011 ▸; Homanen *et al.*, 1996 ▸; Vandenburgh *et al.*, 2008 ▸), whereas the Ru—N—C bond angles (for N-ligating examples) are in the range 159–179° (Brewster *et al.*, 2011 ▸; Cadranel *et al.*, 2012 ▸; Shklover *et al.*, 2002 ▸; Vandenburgh *et al.*, 2008 ▸; Zakeeruddin *et al.*, 1997 ▸). In the present cases, the Ru—*X*—C bond angles of compounds (I) and (II) are 175.6 (3) and 166.03 (19)°, respectively, indicating that the Ru^II^ atoms in both compounds exhibit an N-coordination.

The bond length between the Ru^II^ atom and the nitro­gen atom in (I) [2.105 (3) Å] is slightly longer than that of (II) [2.069 (2) Å]. In contrast, the N≡C bond length in (I) [1.116 (5) Å] is shorter than that of (II) [1.160 (3) Å]. The terminal C—S distance [(I): 1.637 (4) Å, (II): 1.647 (2) Å] and the N—C—S bond angle [(I): 178.2 (4)°, (II): 179.0 (2)°] are similar. These data are in agreement with those of the related polypyridyl complexes containing N-bound {Ru^II^–NCS}^+^ moieties (Brewster *et al.*, 2011 ▸; Cadranel *et al.*, 2012 ▸; Shklover *et al.*, 2002 ▸; Vandenburgh *et al.*, 2008 ▸; Zakeeruddin *et al.*, 1997 ▸).

## 3. Supra­molecular features

**Figure 3:**
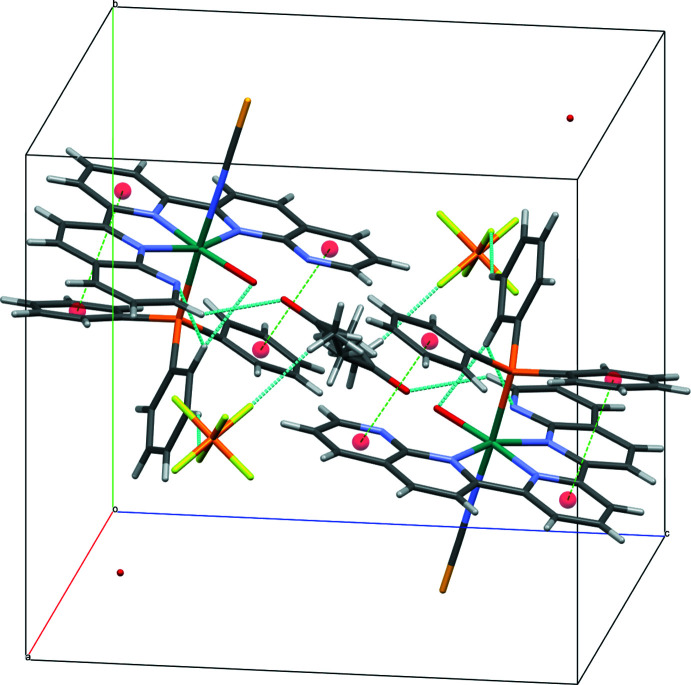
The crystal packing of compound (I) with hydrogen bonds (blue; for numerical details, see Table 1 ▸) and π–π contacts (green) shown as dashed lines. Ring centroids are shown as red spheres.

Additional solvent mol­ecules are incorporated in the crystal structure of (I), *i.e*., a water mol­ecule and a disordered acetone mol­ecule (occupancy 0.5) per formula unit. Apart from Coulombic forces, there are weak C—H⋯F hydrogen bonds between the complex cation and the PF_6_^−^ anion (Table 1 ▸) and the acetone mol­ecule [O1⋯O2 = 2.87 (1) Å]. These inter­actions contribute to the stabilization of the packing and formation of a three-dimensional supra­molecular structure (Fig. 3 ▸).

**Figure 4:**
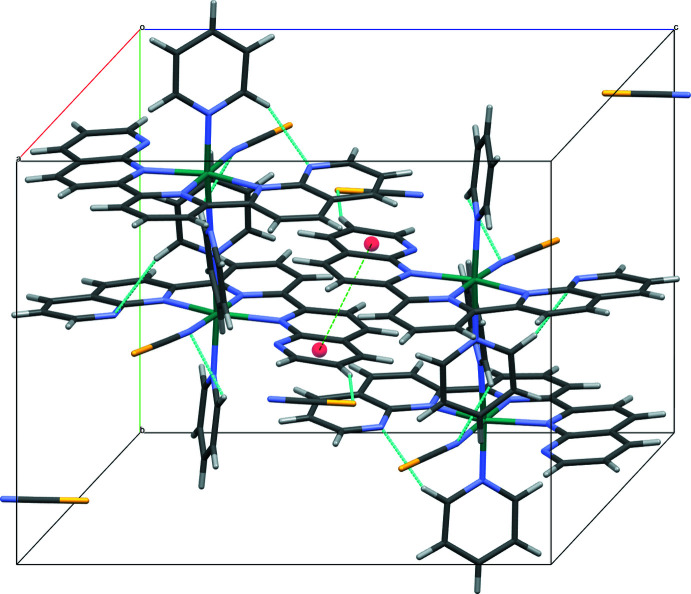
The crystal packing of compound (II) with hydrogen bonds (blue; for numerical details, see Table 2 ▸) and π–π contacts (green) shown as dashed lines. Ring centroids are shown as red spheres.

In the crystal structure of (II), weak C—H⋯*X* (*X* = N or S) hydrogen-bonding inter­actions exist between the complex cation and the NCS^−^ anion (Table 2 ▸) along with the intra­molecular hydrogen bonds. Additional π–π inter­actions [*Cg*5⋯*Cg*5^i^ = 4.0093 (15) Å; *Cg*5 is the centroid of the N5/C17–C21 ring; symmetry code: (i) 1 − *x*, 1 − *y*, 1 − *z*] with a centroid slippage of 1.263 Å for *Cg*5⋯*Cg*5^i^ are present. The slippage angle *β* is 18.4° for *Cg*5⋯*Cg*5^i^. These inter­actions lead to the formation of a three-dimensional network structure (Fig. 4 ▸).

## 4. Database survey

Some crystal structures of Ru^II^ complexes with both N-coordinating thio­cyanato and tridentate terpyridine derivative ligands (tpy*R*) of the form [Ru(tpy*R*)(*N*CS)*L*_2_]^*n*^ (*R* = various substituents, *L* = pyridyl or NCS ligands) have been reported, as revealed by a search of the Cambridge Crystal Structure Database (CSD, version 5.42, update September 2021; Groom *et al.*, 2016 ▸), including refcodes NAMCEL (Brewster *et al.*, 2011 ▸), CAQRAP (Cadranel *et al.*, 2012 ▸), MIXGOP01 (Shklover *et al.*, 2002 ▸), and NUMBOM (Zakeeruddin *et al.*, 1997 ▸). In contrast, for NAMCIP (Brewster *et al.*, 2011 ▸), TORMIW (Homanen *et al.*, 1996 ▸) and EGAYUH (Vandenburgh *et al.*, 2008 ▸) S-coordinating thio­cyanato ligands in polypyridyl­ruthenium(II) complexes were reported.

## 5. Synthesis and crystallization

A methano­lic solution (40 ml) containing [Ru(dnp)(PPh_3_)_2_(H_2_O)](PF_6_)_2_ (50 mg, 0.039 mmol) (Oyama *et al.*, 2013 ▸) and 1.1 eq. of NaSCN (10 mg) was heated under reflux for 30 min. The volume was reduced to 5 ml, and a saturated solution of KPF_6_ was added. The resulting solid was filtered and washed sequentially with water and diethyl ether. The yield was 32 mg (69%). Crystals suitable for use in X-ray diffraction (XRD) studies were grown by vapor diffusion of diethyl ether into an acetone solution of (I). Fourier transform infrared (FTIR) spectroscopy using a KBr pellet showed ν_CN_ at 2130 cm^−1^.

For the synthesis of compound (II), a methano­lic solution (20 ml) containing [Ru(dnp)(py)_2_(H_2_O)](PF_6_)_2_ (25 mg, 0.028 mmol) (Oyama *et al.*, 2013 ▸) and 2.2 eq. of NaSCN (5 mg) was heated under reflux for 30 min. The reaction mixture was reduced to 3 ml. The addition of diethyl ether (5 ml) to the solution resulted in the formation of a precipitate of (II). The crude product was purified by column chromatography on Al_2_O_3_ (eluent: acetone). The yield was 9 mg (40%). Single crystals suitable for XRD studies were obtained by recrystallization from acetone. FTIR using a KBr pellet showed ν_CN_ at 2121 (ligand) and 2055 cm^−1^ (counter-ion).

## 6. Refinement

Table 3 ▸ lists the crystal data, data collection, and structure refinement details. All hydrogen atoms were placed at calculated positions [C—H = 0.93 or 0.96 Å in (I), C—H = 0.95 Å in (II)] and refined using a riding model with *U*_iso_(H) = 1.2*U*_eq_(C). The acetone solvent mol­ecule in (I) (C41–C43, O2) is disordered over an inversion center and was refined with an occupancy of 0.5. The oxygen atom of the solvent water mol­ecule (O3) was refined with an isotropic displacement parameter. H atoms of the coordinating and the solvate water mol­ecules could not be localized from difference-Fourier maps. Therefore, they are not part of the model but part of the formula.

## Supplementary Material

Crystal structure: contains datablock(s) global, I, II. DOI: 10.1107/S2056989022004443/wm5641sup1.cif

Structure factors: contains datablock(s) I. DOI: 10.1107/S2056989022004443/wm5641Isup2.hkl

Structure factors: contains datablock(s) II. DOI: 10.1107/S2056989022004443/wm5641IIsup3.hkl

CCDC references: 2168839, 2168838

Additional supporting information:  crystallographic information; 3D view; checkCIF report

## Figures and Tables

**Figure 2:**
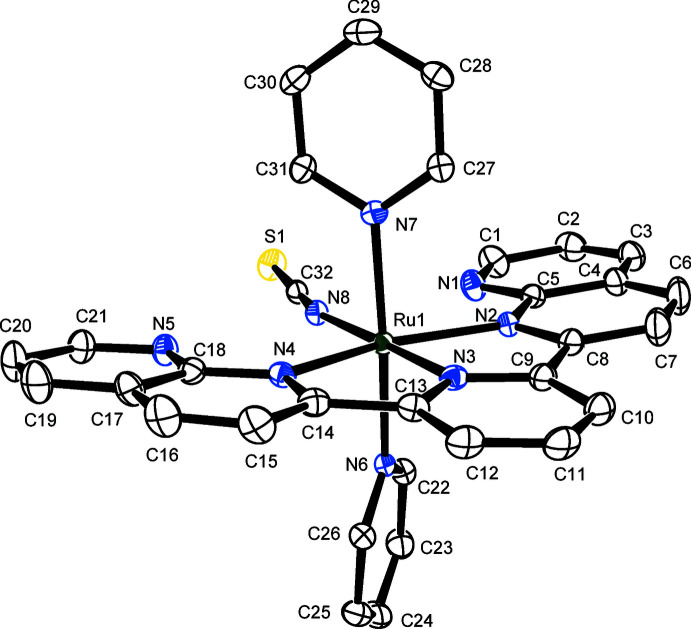
Mol­ecular structure of the complex cation in (II), with atom labels and displacement ellipsoids for non-H atoms drawn at the 50% probability level.

**Table 1:** Hydrogen-bond geometry (Å, °) for (I)

*D*—H⋯*A*	*D*—H	H⋯*A*	*D*⋯*A*	*D*—H⋯*A*
C10—H6⋯F5^i^	0.93	2.45	3.369 (6)	170
C15—H9⋯F2	0.93	2.45	3.345 (6)	162
C21—H13⋯O2	0.93	2.59	3.213 (14)	124
C24—H14⋯O1	0.93	2.43	3.210 (5)	141
C24—H14⋯N5	0.93	2.43	3.144 (6)	134
C25—H15⋯F4^ii^	0.93	2.54	3.347 (7)	145
C41—H30⋯F1^ii^	0.96	2.40	3.26 (3)	150

Symmetry codes: (i) 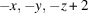 ; (ii) 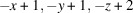 .

**Table 2:** Hydrogen-bond geometry (Å, °) for (II)

*D*—H⋯*A*	*D*—H	H⋯*A*	*D*⋯*A*	*D*—H⋯*A*
C12—H8⋯N9^i^	0.95	2.43	3.305 (5)	152
C20—H12⋯S2^ii^	0.95	2.73	3.629 (3)	159
C22—H14⋯N1	0.95	2.51	3.391 (3)	154
C27—H19⋯S2	0.95	2.76	3.479 (3)	133

Symmetry codes: (i) 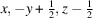 ; (ii) 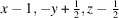 .

**Table 3:** Experimental details

	(I)	(II)
Crystal data		
Chemical formula	[Ru(NCS)(C_21_H_13_N_5_)(C_18_H_15_P)(H_2_O)]PF_6_·0.5C_3_H_6_O·H_2_O	[Ru(NCS)(C_21_H_13_N_5_)(C_5_H_5_N)_2_]NCS
*M*_r_	966.84	710.79
Crystal system, space group	Triclinic, *P* 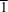	Monoclinic, *P*2_1_/*c*
Temperature (K)	296	93
*a*, *b*, *c* (Å)	9.3699 (2), 15.3897 (4), 16.0267 (4)	12.6556 (10), 14.0986 (7), 17.4421 (14)
α, β, γ (°)	92.6869 (9), 105.1544 (8), 100.0149 (7)	90, 108.535 (3), 90
*V* (Å^3^)	2186.29 (10)	2950.7 (4)
*Z*	2	4
Radiation type	Mo *K*α	Mo *K*α
μ (mm^−1^)	0.55	0.72
Crystal size (mm)	0.20 × 0.15 × 0.10	0.25 × 0.15 × 0.05

Data collection		
Diffractometer	Rigaku R-AXIS RAPID	Rigaku Saturn724
Absorption correction	Multi-scan (*ABSCOR*; Rigaku, 1995 ▸)	Multi-scan (*REQAB*; Rigaku, 1998 ▸)
*T*_min_, *T*_max_	0.750, 0.947	0.927, 0.965
No. of measured, independent and observed [*F* ^2^ > 2.0σ(*F* ^2^)] reflections	34567, 9994, 8406	30135, 6758, 6058
*R*_int_	0.025	0.029
(sin θ/λ)_max_ (Å^−1^)	0.649	0.649

Refinement		
*R*[*F* ^2^ > 2σ(*F* ^2^)], *wR*(*F* ^2^), *S*	0.050, 0.169, 1.08	0.036, 0.091, 1.10
No. of reflections	9994	6758
No. of parameters	554	406
H-atom treatment	H-atom parameters constrained	H-atom parameters constrained
Δρ_max_, Δρ_min_ (e Å^−3^)	1.93, −0.64	1.13, −0.81

Computer programs: *RAPID-AUTO* (Rigaku, 2006 ▸), *CrystalClear* (Rigaku, 2015 ▸), *SIR97* (Altomare *et al.*, 1999 ▸), *SIR92* (Altomare *et al.*, 1993 ▸), *SHELXL2018/3* (Sheldrick, 2015 ▸), *Mercury* (Macrae *et al.*, 2020 ▸), *ORTEP-3 for Windows* (Farrugia, 2012 ▸), *CrystalStructure* (Rigaku, 2019 ▸), *PLATON* (Spek, 2020 ▸) and *publCIF* (Westrip, 2010 ▸).

## supplementary crystallographic information

### cis-Aqua[2,6-bis(1,8-naphthyridin-2-yl)pyridine-κ3N,N',N''](thiocyanato-κN)(triphenylphosphine-κP)ruthenium(II) hexafluoridophosphate–acetone–water (1/0.5/1) (I) Crystal data

**Table:**

[Ru(NCS)(C_21_H_13_N_5_)(C_18_H_15_P)(H_2_O)]PF_6_·0.5C_3_H_6_O·H_2_O	*Z* = 2
*M**_r_* = 966.84	*F*(000) = 980.00
Triclinic, *P*1	*D*_x_ = 1.469 Mg m^−^^3^
*a* = 9.3699 (2) Å	Mo *K*α radiation, λ = 0.71075 Å
*b* = 15.3897 (4) Å	Cell parameters from 29007 reflections
*c* = 16.0267 (4) Å	θ = 3.0–27.5°
α = 92.6869 (9)°	µ = 0.55 mm^−^^1^
β = 105.1544 (8)°	*T* = 296 K
γ = 100.0149 (7)°	Block, purple
*V* = 2186.29 (10) Å^3^	0.20 × 0.15 × 0.10 mm

### cis-Aqua[2,6-bis(1,8-naphthyridin-2-yl)pyridine-κ3N,N',N''](thiocyanato-κN)(triphenylphosphine-κP)ruthenium(II) hexafluoridophosphate–acetone–water (1/0.5/1) (I) Data collection

**Table:**

Rigaku R-AXIS RAPID diffractometer	8406 reflections with *F*^2^ > 2.0σ(*F*^2^)
Detector resolution: 10.000 pixels mm^-1^	*R*_int_ = 0.025
ω scans	θ_max_ = 27.5°, θ_min_ = 3.0°
Absorption correction: multi-scan (ABSCOR; Rigaku, 1995)	*h* = −11→12
*T*_min_ = 0.750, *T*_max_ = 0.947	*k* = −19→19
34567 measured reflections	*l* = −20→20
9994 independent reflections	

### cis-Aqua[2,6-bis(1,8-naphthyridin-2-yl)pyridine-κ3N,N',N''](thiocyanato-κN)(triphenylphosphine-κP)ruthenium(II) hexafluoridophosphate–acetone–water (1/0.5/1) (I) Refinement

**Table:**

Refinement on *F*^2^	Secondary atom site location: difference Fourier map
*R*[*F*^2^ > 2σ(*F*^2^)] = 0.050	Hydrogen site location: inferred from neighbouring sites
*wR*(*F*^2^) = 0.169	H-atom parameters constrained
*S* = 1.08	*w* = 1/[σ^2^(*F*_o_^2^) + (0.1102*P*)^2^ + 1.1681*P*] where *P* = (*F*_o_^2^ + 2*F*_c_^2^)/3
9994 reflections	(Δ/σ)_max_ = 0.001
554 parameters	Δρ_max_ = 1.93 e Å^−^^3^
0 restraints	Δρ_min_ = −0.64 e Å^−^^3^
Primary atom site location: structure-invariant direct methods	

### cis-Aqua[2,6-bis(1,8-naphthyridin-2-yl)pyridine-κ3N,N',N''](thiocyanato-κN)(triphenylphosphine-κP)ruthenium(II) hexafluoridophosphate–acetone–water (1/0.5/1) (I) Special details

**Table:**

Geometry. All esds (except the esd in the dihedral angle between two l.s. planes) are estimated using the full covariance matrix. The cell esds are taken into account individually in the estimation of esds in distances, angles and torsion angles; correlations between esds in cell parameters are only used when they are defined by crystal symmetry. An approximate (isotropic) treatment of cell esds is used for estimating esds involving l.s. planes.
Refinement. Refinement was performed using all reflections. The weighted R-factor (wR) and goodness of fit (S) are based on F^2^. R-factor (gt) are based on F. The threshold expression of F^2^ > 2.0 sigma(F^2^) is used only for calculating R-factor (gt).

### cis-Aqua[2,6-bis(1,8-naphthyridin-2-yl)pyridine-κ3N,N',N''](thiocyanato-κN)(triphenylphosphine-κP)ruthenium(II) hexafluoridophosphate–acetone–water (1/0.5/1) (I) Fractional atomic coordinates and isotropic or equivalent isotropic displacement parameters (Å^2^)

**Table:**

	*x*	*y*	*z*	*U*_iso_*/*U*_eq_	Occ. (<1)
Ru1	0.20144 (3)	0.23092 (2)	0.71810 (2)	0.03823 (11)	
S1	0.54439 (14)	0.03779 (10)	0.68390 (13)	0.0978 (5)	
P1	0.02612 (10)	0.31990 (6)	0.72697 (6)	0.0440 (2)	
P2	0.28893 (14)	0.24126 (7)	1.22614 (7)	0.0588 (3)	
F1	0.3298 (6)	0.3196 (2)	1.3006 (2)	0.1247 (15)	
F2	0.2484 (4)	0.16220 (19)	1.15150 (19)	0.0894 (9)	
F3	0.2730 (5)	0.3108 (2)	1.1558 (2)	0.1043 (11)	
F4	0.4608 (4)	0.2498 (3)	1.2296 (3)	0.1163 (13)	
F5	0.3020 (4)	0.1719 (2)	1.2970 (2)	0.0948 (10)	
F6	0.1186 (4)	0.2322 (3)	1.2222 (3)	0.1110 (12)	
O1	0.2872 (3)	0.31914 (19)	0.63264 (18)	0.0589 (7)	
O3	−0.4347 (17)	0.0497 (10)	0.1027 (10)	0.344 (7)*	
N1	0.0916 (4)	0.2097 (2)	0.5019 (2)	0.0575 (8)	
N2	0.0397 (3)	0.14350 (19)	0.62044 (18)	0.0410 (6)	
N3	0.1213 (3)	0.14515 (18)	0.78696 (18)	0.0415 (6)	
N4	0.3425 (3)	0.27857 (19)	0.84298 (19)	0.0435 (6)	
N5	0.4997 (4)	0.3928 (2)	0.8046 (2)	0.0578 (8)	
N6	0.3590 (3)	0.1529 (2)	0.7042 (2)	0.0508 (7)	
C1	0.0597 (6)	0.2151 (3)	0.4174 (3)	0.0699 (13)	
H1	0.119932	0.258875	0.396905	0.084*	
C2	−0.0596 (6)	0.1587 (3)	0.3564 (3)	0.0666 (11)	
H2	−0.078108	0.165630	0.297422	0.080*	
C3	−0.1474 (5)	0.0940 (3)	0.3851 (3)	0.0626 (10)	
H3	−0.227518	0.055966	0.346082	0.075*	
C4	−0.1156 (4)	0.0850 (3)	0.4755 (2)	0.0504 (8)	
C5	0.0039 (4)	0.1462 (2)	0.5316 (2)	0.0462 (7)	
C6	−0.1963 (4)	0.0176 (3)	0.5117 (3)	0.0562 (9)	
H4	−0.275944	−0.023686	0.476010	0.067*	
C7	−0.1565 (4)	0.0136 (2)	0.5989 (3)	0.0505 (8)	
H5	−0.207602	−0.031113	0.623294	0.061*	
C8	−0.0377 (4)	0.0773 (2)	0.6521 (2)	0.0428 (7)	
C9	0.0081 (4)	0.0765 (2)	0.7467 (2)	0.0459 (7)	
C10	−0.0498 (5)	0.0139 (3)	0.7941 (3)	0.0644 (11)	
H6	−0.128217	−0.032599	0.766539	0.077*	
C11	0.0111 (6)	0.0216 (3)	0.8841 (3)	0.0817 (16)	
H7	−0.026723	−0.019790	0.917268	0.098*	
C12	0.1283 (6)	0.0913 (3)	0.9237 (3)	0.0760 (14)	
H8	0.170109	0.096773	0.983515	0.091*	
C13	0.1822 (4)	0.1523 (3)	0.8740 (2)	0.0516 (8)	
C14	0.3075 (4)	0.2288 (3)	0.9051 (2)	0.0511 (8)	
C15	0.3825 (5)	0.2494 (3)	0.9935 (3)	0.0679 (12)	
H9	0.354640	0.213987	1.034253	0.081*	
C16	0.4958 (6)	0.3212 (3)	1.0194 (3)	0.0720 (13)	
H10	0.543541	0.336458	1.078127	0.086*	
C17	0.5405 (5)	0.3718 (3)	0.9579 (3)	0.0579 (10)	
C18	0.4624 (4)	0.3486 (2)	0.8687 (2)	0.0467 (7)	
C19	0.6592 (6)	0.4471 (3)	0.9779 (3)	0.0787 (14)	
H11	0.713260	0.465186	1.035344	0.094*	
C20	0.6933 (6)	0.4921 (3)	0.9140 (4)	0.0817 (15)	
H12	0.769460	0.542262	0.926604	0.098*	
C21	0.6114 (5)	0.4620 (3)	0.8266 (4)	0.0720 (13)	
H13	0.637933	0.492736	0.782642	0.086*	
C22	0.4359 (4)	0.1070 (3)	0.6970 (3)	0.0542 (9)	
C23	0.0862 (5)	0.4408 (2)	0.7478 (3)	0.0543 (9)	
C24	0.2210 (5)	0.4838 (3)	0.7372 (3)	0.0607 (10)	
H14	0.286908	0.451352	0.722033	0.073*	
C25	0.2587 (7)	0.5761 (3)	0.7493 (4)	0.0816 (15)	
H15	0.350439	0.604667	0.742700	0.098*	
C26	0.1643 (9)	0.6245 (3)	0.7705 (4)	0.099 (2)	
H16	0.189677	0.686032	0.777275	0.119*	
C27	0.0335 (10)	0.5829 (4)	0.7817 (5)	0.115 (2)	
H17	−0.031213	0.616375	0.796792	0.137*	
C28	−0.0074 (8)	0.4916 (4)	0.7713 (5)	0.0931 (18)	
H18	−0.098155	0.464252	0.780230	0.112*	
C29	−0.1240 (5)	0.3099 (3)	0.6248 (3)	0.0638 (11)	
C30	−0.2398 (7)	0.2426 (5)	0.5962 (5)	0.105 (2)	
H19	−0.253912	0.198479	0.632581	0.126*	
C31	−0.3409 (10)	0.2352 (6)	0.5141 (6)	0.139 (3)	
H20	−0.425729	0.190055	0.499432	0.167*	
C32	−0.3191 (12)	0.2902 (7)	0.4576 (5)	0.151 (4)	
H21	−0.387200	0.285053	0.402952	0.181*	
C33	−0.1974 (12)	0.3538 (9)	0.4799 (5)	0.174 (5)	
H22	−0.177257	0.392207	0.439500	0.209*	
C34	−0.0982 (8)	0.3640 (7)	0.5639 (4)	0.135 (3)	
H23	−0.012944	0.408900	0.577956	0.162*	
C35	−0.0579 (4)	0.2952 (3)	0.8167 (2)	0.0499 (8)	
C36	0.0264 (6)	0.3277 (4)	0.8996 (3)	0.0752 (13)	
H24	0.118188	0.366055	0.907513	0.090*	
C37	−0.0212 (7)	0.3050 (5)	0.9710 (3)	0.0932 (18)	
H25	0.038846	0.328108	1.026088	0.112*	
C38	−0.1535 (7)	0.2498 (4)	0.9626 (4)	0.0909 (17)	
H26	−0.184622	0.233571	1.011189	0.109*	
C39	−0.2415 (8)	0.2180 (6)	0.8801 (5)	0.131 (3)	
H27	−0.334539	0.181065	0.872839	0.158*	
C40	−0.1934 (7)	0.2401 (5)	0.8078 (4)	0.111 (3)	
H28	−0.253921	0.217311	0.752646	0.133*	
O2	0.5573 (15)	0.4320 (11)	0.6201 (8)	0.162 (6)	0.5
C41	0.688 (3)	0.5339 (16)	0.5506 (16)	0.156 (8)	0.5
H29	0.663565	0.561416	0.497545	0.187*	0.5
H30	0.723079	0.578661	0.598965	0.187*	0.5
H31	0.765916	0.500822	0.549928	0.187*	0.5
C42	0.554 (2)	0.4738 (11)	0.5589 (12)	0.124 (5)	0.5
C43	0.414 (4)	0.454 (3)	0.480 (2)	0.28 (3)	0.5
H32	0.429253	0.493342	0.436933	0.335*	0.5
H33	0.399037	0.393942	0.456380	0.335*	0.5
H34	0.327501	0.463494	0.498052	0.335*	0.5

### cis-Aqua[2,6-bis(1,8-naphthyridin-2-yl)pyridine-κ3N,N',N''](thiocyanato-κN)(triphenylphosphine-κP)ruthenium(II) hexafluoridophosphate–acetone–water (1/0.5/1) (I) Atomic displacement parameters (Å^2^)

**Table:**

	*U*^11^	*U*^22^	*U*^33^	*U*^12^	*U*^13^	*U*^23^
Ru1	0.03849 (16)	0.04036 (16)	0.03340 (15)	−0.00234 (10)	0.01317 (10)	−0.00263 (10)
S1	0.0506 (6)	0.0878 (9)	0.1451 (14)	0.0143 (6)	0.0182 (8)	−0.0414 (9)
P1	0.0419 (5)	0.0462 (5)	0.0400 (4)	0.0036 (3)	0.0091 (3)	−0.0047 (3)
P2	0.0728 (7)	0.0505 (5)	0.0496 (5)	−0.0018 (5)	0.0197 (5)	0.0032 (4)
F1	0.203 (5)	0.074 (2)	0.084 (2)	−0.002 (2)	0.041 (3)	−0.0228 (17)
F2	0.124 (3)	0.0687 (17)	0.0681 (17)	−0.0153 (16)	0.0381 (17)	−0.0117 (14)
F3	0.154 (3)	0.077 (2)	0.084 (2)	0.015 (2)	0.038 (2)	0.0341 (17)
F4	0.070 (2)	0.112 (3)	0.161 (4)	−0.0060 (18)	0.036 (2)	0.012 (3)
F5	0.131 (3)	0.0773 (19)	0.0681 (18)	0.0050 (18)	0.0212 (18)	0.0231 (15)
F6	0.085 (2)	0.138 (3)	0.128 (3)	0.028 (2)	0.052 (2)	0.033 (3)
O1	0.0666 (17)	0.0576 (16)	0.0482 (15)	−0.0099 (13)	0.0226 (13)	0.0040 (12)
N1	0.072 (2)	0.0569 (19)	0.0380 (15)	−0.0108 (15)	0.0213 (15)	−0.0055 (13)
N2	0.0405 (14)	0.0444 (14)	0.0352 (13)	−0.0002 (11)	0.0125 (11)	−0.0058 (11)
N3	0.0465 (15)	0.0401 (14)	0.0353 (13)	0.0000 (11)	0.0129 (11)	−0.0014 (11)
N4	0.0427 (15)	0.0453 (15)	0.0388 (14)	0.0008 (11)	0.0108 (11)	−0.0025 (11)
N5	0.0460 (17)	0.0592 (19)	0.059 (2)	−0.0063 (14)	0.0095 (14)	0.0053 (15)
N6	0.0459 (16)	0.0557 (17)	0.0500 (17)	0.0058 (13)	0.0163 (13)	−0.0058 (14)
C1	0.089 (3)	0.073 (3)	0.040 (2)	−0.013 (2)	0.024 (2)	−0.0009 (18)
C2	0.081 (3)	0.076 (3)	0.0365 (19)	0.002 (2)	0.0151 (19)	−0.0025 (18)
C3	0.061 (2)	0.076 (3)	0.0411 (19)	−0.001 (2)	0.0089 (17)	−0.0093 (18)
C4	0.0473 (19)	0.060 (2)	0.0391 (17)	0.0008 (16)	0.0126 (15)	−0.0078 (15)
C5	0.0494 (19)	0.0501 (18)	0.0373 (16)	0.0006 (14)	0.0166 (14)	−0.0062 (14)
C6	0.049 (2)	0.059 (2)	0.051 (2)	−0.0070 (16)	0.0116 (16)	−0.0128 (17)
C7	0.0495 (19)	0.0469 (18)	0.051 (2)	−0.0047 (15)	0.0185 (16)	−0.0067 (15)
C8	0.0438 (17)	0.0411 (16)	0.0413 (17)	−0.0008 (13)	0.0151 (14)	−0.0034 (13)
C9	0.0486 (18)	0.0439 (17)	0.0422 (17)	−0.0035 (14)	0.0165 (14)	−0.0022 (14)
C10	0.075 (3)	0.057 (2)	0.051 (2)	−0.0184 (19)	0.0198 (19)	0.0019 (18)
C11	0.100 (4)	0.079 (3)	0.050 (2)	−0.032 (3)	0.023 (2)	0.012 (2)
C12	0.095 (3)	0.079 (3)	0.039 (2)	−0.024 (3)	0.017 (2)	0.0074 (19)
C13	0.059 (2)	0.055 (2)	0.0360 (17)	−0.0048 (16)	0.0148 (15)	0.0002 (14)
C14	0.059 (2)	0.055 (2)	0.0346 (16)	−0.0044 (16)	0.0138 (15)	−0.0005 (14)
C15	0.072 (3)	0.079 (3)	0.0388 (19)	−0.012 (2)	0.0099 (18)	0.0017 (19)
C16	0.078 (3)	0.080 (3)	0.039 (2)	−0.007 (2)	0.0000 (19)	−0.0096 (19)
C17	0.051 (2)	0.059 (2)	0.051 (2)	−0.0018 (17)	0.0020 (16)	−0.0105 (17)
C18	0.0393 (17)	0.0478 (18)	0.0475 (19)	0.0015 (13)	0.0085 (14)	−0.0042 (14)
C19	0.073 (3)	0.070 (3)	0.067 (3)	−0.014 (2)	−0.006 (2)	−0.010 (2)
C20	0.064 (3)	0.068 (3)	0.086 (4)	−0.023 (2)	−0.001 (2)	0.002 (3)
C21	0.056 (2)	0.064 (3)	0.083 (3)	−0.0117 (19)	0.011 (2)	0.011 (2)
C22	0.0392 (18)	0.060 (2)	0.057 (2)	−0.0043 (16)	0.0135 (16)	−0.0141 (17)
C23	0.067 (2)	0.0447 (19)	0.049 (2)	0.0117 (17)	0.0121 (17)	0.0022 (15)
C24	0.062 (2)	0.050 (2)	0.059 (2)	0.0021 (17)	0.0035 (19)	0.0030 (17)
C25	0.093 (4)	0.053 (3)	0.076 (3)	−0.009 (2)	−0.001 (3)	0.008 (2)
C26	0.156 (6)	0.043 (2)	0.083 (4)	0.019 (3)	0.006 (4)	0.004 (2)
C27	0.155 (7)	0.066 (4)	0.138 (6)	0.049 (4)	0.050 (6)	−0.001 (4)
C28	0.104 (4)	0.066 (3)	0.128 (5)	0.029 (3)	0.058 (4)	0.004 (3)
C29	0.060 (2)	0.075 (3)	0.048 (2)	0.018 (2)	−0.0011 (18)	−0.0085 (19)
C30	0.086 (4)	0.104 (5)	0.091 (4)	−0.004 (3)	−0.019 (3)	−0.006 (3)
C31	0.107 (6)	0.145 (7)	0.109 (6)	−0.002 (5)	−0.044 (5)	−0.021 (5)
C32	0.164 (9)	0.178 (9)	0.077 (5)	0.075 (7)	−0.047 (5)	−0.031 (5)
C33	0.148 (8)	0.297 (15)	0.056 (4)	0.035 (9)	−0.007 (5)	0.036 (6)
C34	0.092 (5)	0.244 (10)	0.055 (3)	0.015 (5)	0.003 (3)	0.036 (5)
C35	0.0435 (18)	0.055 (2)	0.0495 (19)	0.0011 (14)	0.0166 (15)	−0.0077 (15)
C36	0.068 (3)	0.095 (3)	0.050 (2)	−0.015 (2)	0.017 (2)	−0.008 (2)
C37	0.089 (4)	0.129 (5)	0.048 (3)	−0.010 (3)	0.018 (2)	−0.010 (3)
C38	0.099 (4)	0.107 (4)	0.074 (3)	−0.005 (3)	0.053 (3)	0.001 (3)
C39	0.113 (5)	0.165 (7)	0.101 (5)	−0.066 (5)	0.071 (4)	−0.037 (5)
C40	0.088 (4)	0.151 (6)	0.067 (3)	−0.060 (4)	0.034 (3)	−0.032 (3)
O2	0.146 (10)	0.222 (15)	0.095 (8)	−0.035 (10)	0.035 (7)	0.026 (9)
C41	0.21 (3)	0.121 (15)	0.144 (19)	0.027 (17)	0.064 (18)	0.014 (14)
C42	0.158 (16)	0.094 (10)	0.107 (12)	−0.008 (10)	0.040 (11)	−0.005 (9)
C43	0.26 (5)	0.30 (5)	0.18 (3)	−0.06 (3)	−0.07 (3)	0.11 (3)

### cis-Aqua[2,6-bis(1,8-naphthyridin-2-yl)pyridine-κ3N,N',N''](thiocyanato-κN)(triphenylphosphine-κP)ruthenium(II) hexafluoridophosphate–acetone–water (1/0.5/1) (I) Geometric parameters (Å, º)

**Table:**

Ru1—N3	1.936 (3)	C16—C17	1.388 (7)
Ru1—N2	2.100 (3)	C16—H10	0.9300
Ru1—N4	2.105 (3)	C17—C19	1.419 (6)
Ru1—N6	2.105 (3)	C17—C18	1.421 (5)
Ru1—O1	2.176 (3)	C19—C20	1.338 (8)
Ru1—P1	2.3409 (9)	C19—H11	0.9300
S1—C22	1.637 (4)	C20—C21	1.421 (7)
P1—C35	1.836 (4)	C20—H12	0.9300
P1—C23	1.836 (4)	C21—H13	0.9300
P1—C29	1.839 (4)	C23—C24	1.377 (6)
P2—F6	1.562 (4)	C23—C28	1.380 (7)
P2—F1	1.577 (3)	C24—C25	1.393 (6)
P2—F4	1.579 (4)	C24—H14	0.9300
P2—F2	1.588 (3)	C25—C26	1.346 (9)
P2—F3	1.588 (3)	C25—H15	0.9300
P2—F5	1.590 (3)	C26—C27	1.340 (10)
N1—C1	1.319 (5)	C26—H16	0.9300
N1—C5	1.350 (5)	C27—C28	1.381 (8)
N2—C8	1.345 (4)	C27—H17	0.9300
N2—C5	1.379 (4)	C28—H18	0.9300
N3—C13	1.353 (4)	C29—C30	1.328 (7)
N3—C9	1.359 (4)	C29—C34	1.355 (9)
N4—C14	1.356 (5)	C30—C31	1.392 (9)
N4—C18	1.374 (4)	C30—H19	0.9300
N5—C21	1.318 (5)	C31—C32	1.298 (13)
N5—C18	1.346 (5)	C31—H20	0.9300
N6—C22	1.116 (5)	C32—C33	1.324 (15)
C1—C2	1.403 (6)	C32—H21	0.9300
C1—H1	0.9300	C33—C34	1.406 (9)
C2—C3	1.355 (6)	C33—H22	0.9300
C2—H2	0.9300	C34—H23	0.9300
C3—C4	1.419 (5)	C35—C40	1.368 (6)
C3—H3	0.9300	C35—C36	1.376 (6)
C4—C5	1.407 (5)	C36—C37	1.370 (7)
C4—C6	1.413 (6)	C36—H24	0.9300
C6—C7	1.356 (6)	C37—C38	1.346 (8)
C6—H4	0.9300	C37—H25	0.9300
C7—C8	1.404 (5)	C38—C39	1.377 (9)
C7—H5	0.9300	C38—H26	0.9300
C8—C9	1.465 (5)	C39—C40	1.385 (8)
C9—C10	1.379 (5)	C39—H27	0.9300
C10—C11	1.397 (6)	C40—H28	0.9300
C10—H6	0.9300	O2—C42	1.19 (2)
C11—C12	1.386 (6)	C41—C42	1.47 (3)
C11—H7	0.9300	C41—H29	0.9600
C12—C13	1.374 (5)	C41—H30	0.9600
C12—H8	0.9300	C41—H31	0.9600
C13—C14	1.474 (5)	C42—C43	1.53 (3)
C14—C15	1.400 (5)	C43—H32	0.9600
C15—C16	1.356 (6)	C43—H33	0.9600
C15—H9	0.9300	C43—H34	0.9600
			
N3—Ru1—N2	79.15 (11)	C16—C15—H9	120.2
N3—Ru1—N4	79.47 (11)	C14—C15—H9	120.2
N2—Ru1—N4	158.24 (12)	C15—C16—C17	119.8 (4)
N3—Ru1—N6	90.12 (13)	C15—C16—H10	120.1
N2—Ru1—N6	87.81 (11)	C17—C16—H10	120.1
N4—Ru1—N6	88.21 (12)	C16—C17—C19	124.3 (4)
N3—Ru1—O1	175.58 (11)	C16—C17—C18	118.8 (4)
N2—Ru1—O1	96.97 (11)	C19—C17—C18	116.8 (4)
N4—Ru1—O1	104.23 (11)	N5—C18—N4	115.9 (3)
N6—Ru1—O1	87.59 (13)	N5—C18—C17	122.9 (3)
N3—Ru1—P1	92.21 (9)	N4—C18—C17	121.2 (3)
N2—Ru1—P1	91.23 (8)	C20—C19—C17	119.9 (4)
N4—Ru1—P1	93.61 (8)	C20—C19—H11	120.0
N6—Ru1—P1	177.27 (9)	C17—C19—H11	120.0
O1—Ru1—P1	89.98 (9)	C19—C20—C21	119.0 (4)
C35—P1—C23	100.93 (18)	C19—C20—H12	120.5
C35—P1—C29	109.5 (2)	C21—C20—H12	120.5
C23—P1—C29	101.0 (2)	N5—C21—C20	123.5 (5)
C35—P1—Ru1	112.05 (13)	N5—C21—H13	118.3
C23—P1—Ru1	120.14 (14)	C20—C21—H13	118.3
C29—P1—Ru1	112.01 (15)	N6—C22—S1	178.2 (4)
F6—P2—F1	89.9 (3)	C24—C23—C28	118.1 (4)
F6—P2—F4	179.6 (3)	C24—C23—P1	121.8 (3)
F1—P2—F4	90.6 (3)	C28—C23—P1	120.1 (4)
F6—P2—F2	90.3 (2)	C23—C24—C25	119.9 (5)
F1—P2—F2	179.7 (3)	C23—C24—H14	120.0
F4—P2—F2	89.3 (2)	C25—C24—H14	120.0
F6—P2—F3	90.5 (2)	C26—C25—C24	121.1 (6)
F1—P2—F3	89.7 (2)	C26—C25—H15	119.5
F4—P2—F3	89.5 (2)	C24—C25—H15	119.5
F2—P2—F3	90.49 (19)	C27—C26—C25	119.3 (5)
F6—P2—F5	88.4 (2)	C27—C26—H16	120.4
F1—P2—F5	89.9 (2)	C25—C26—H16	120.4
F4—P2—F5	91.5 (2)	C26—C27—C28	121.5 (6)
F2—P2—F5	89.90 (18)	C26—C27—H17	119.2
F3—P2—F5	178.9 (2)	C28—C27—H17	119.2
C1—N1—C5	118.0 (3)	C23—C28—C27	120.1 (6)
C8—N2—C5	118.0 (3)	C23—C28—H18	119.9
C8—N2—Ru1	112.9 (2)	C27—C28—H18	119.9
C5—N2—Ru1	129.1 (2)	C30—C29—C34	114.8 (6)
C13—N3—C9	120.8 (3)	C30—C29—P1	126.1 (5)
C13—N3—Ru1	119.8 (2)	C34—C29—P1	117.3 (4)
C9—N3—Ru1	119.4 (2)	C29—C30—C31	122.9 (7)
C14—N4—C18	117.6 (3)	C29—C30—H19	118.6
C14—N4—Ru1	112.3 (2)	C31—C30—H19	118.6
C18—N4—Ru1	130.1 (2)	C32—C31—C30	121.3 (8)
C21—N5—C18	117.8 (4)	C32—C31—H20	119.4
C22—N6—Ru1	175.6 (3)	C30—C31—H20	119.4
N1—C1—C2	123.9 (4)	C31—C32—C33	118.3 (7)
N1—C1—H1	118.0	C31—C32—H21	120.9
C2—C1—H1	118.0	C33—C32—H21	120.9
C3—C2—C1	118.7 (4)	C32—C33—C34	120.8 (10)
C3—C2—H2	120.6	C32—C33—H22	119.6
C1—C2—H2	120.6	C34—C33—H22	119.6
C2—C3—C4	119.2 (4)	C29—C34—C33	121.4 (9)
C2—C3—H3	120.4	C29—C34—H23	119.3
C4—C3—H3	120.4	C33—C34—H23	119.3
C5—C4—C6	118.6 (3)	C40—C35—C36	117.2 (4)
C5—C4—C3	117.8 (4)	C40—C35—P1	124.4 (3)
C6—C4—C3	123.6 (4)	C36—C35—P1	118.0 (3)
N1—C5—N2	116.2 (3)	C37—C36—C35	121.8 (4)
N1—C5—C4	122.3 (3)	C37—C36—H24	119.1
N2—C5—C4	121.5 (3)	C35—C36—H24	119.1
C7—C6—C4	119.4 (3)	C38—C37—C36	121.1 (5)
C7—C6—H4	120.3	C38—C37—H25	119.4
C4—C6—H4	120.3	C36—C37—H25	119.4
C6—C7—C8	119.7 (3)	C37—C38—C39	118.1 (5)
C6—C7—H5	120.1	C37—C38—H26	120.9
C8—C7—H5	120.1	C39—C38—H26	120.9
N2—C8—C7	122.7 (3)	C38—C39—C40	121.0 (5)
N2—C8—C9	115.5 (3)	C38—C39—H27	119.5
C7—C8—C9	121.8 (3)	C40—C39—H27	119.5
N3—C9—C10	120.6 (3)	C35—C40—C39	120.6 (5)
N3—C9—C8	113.0 (3)	C35—C40—H28	119.7
C10—C9—C8	126.4 (3)	C39—C40—H28	119.7
C9—C10—C11	118.9 (4)	C42—C41—H29	109.5
C9—C10—H6	120.5	C42—C41—H30	109.5
C11—C10—H6	120.5	H29—C41—H30	109.5
C12—C11—C10	119.5 (4)	C42—C41—H31	109.5
C12—C11—H7	120.2	H29—C41—H31	109.5
C10—C11—H7	120.2	H30—C41—H31	109.5
C13—C12—C11	119.6 (4)	O2—C42—C41	121 (2)
C13—C12—H8	120.2	O2—C42—C43	120 (2)
C11—C12—H8	120.2	C41—C42—C43	118 (2)
N3—C13—C12	120.6 (3)	C42—C43—H32	109.5
N3—C13—C14	112.9 (3)	C42—C43—H33	109.5
C12—C13—C14	126.6 (3)	H32—C43—H33	109.5
N4—C14—C15	122.6 (3)	C42—C43—H34	109.5
N4—C14—C13	115.7 (3)	H32—C43—H34	109.5
C15—C14—C13	121.7 (3)	H33—C43—H34	109.5
C16—C15—C14	119.7 (4)		
			
C5—N1—C1—C2	−0.1 (8)	C21—N5—C18—C17	−1.8 (6)
N1—C1—C2—C3	−0.7 (8)	C14—N4—C18—N5	175.8 (3)
C1—C2—C3—C4	−0.3 (7)	Ru1—N4—C18—N5	−2.1 (5)
C2—C3—C4—C5	2.0 (7)	C14—N4—C18—C17	−4.3 (5)
C2—C3—C4—C6	−177.3 (4)	Ru1—N4—C18—C17	177.8 (3)
C1—N1—C5—N2	−179.2 (4)	C16—C17—C18—N5	−178.7 (4)
C1—N1—C5—C4	1.9 (6)	C19—C17—C18—N5	2.1 (6)
C8—N2—C5—N1	−175.2 (3)	C16—C17—C18—N4	1.5 (6)
Ru1—N2—C5—N1	5.5 (5)	C19—C17—C18—N4	−177.8 (4)
C8—N2—C5—C4	3.6 (5)	C16—C17—C19—C20	−179.6 (5)
Ru1—N2—C5—C4	−175.6 (3)	C18—C17—C19—C20	−0.4 (8)
C6—C4—C5—N1	176.5 (4)	C17—C19—C20—C21	−1.4 (9)
C3—C4—C5—N1	−2.9 (6)	C18—N5—C21—C20	−0.2 (8)
C6—C4—C5—N2	−2.3 (6)	C19—C20—C21—N5	1.8 (9)
C3—C4—C5—N2	178.3 (4)	C35—P1—C23—C24	140.2 (4)
C5—C4—C6—C7	−0.2 (6)	C29—P1—C23—C24	−107.1 (4)
C3—C4—C6—C7	179.2 (4)	Ru1—P1—C23—C24	16.6 (4)
C4—C6—C7—C8	1.2 (6)	C35—P1—C23—C28	−42.5 (5)
C5—N2—C8—C7	−2.6 (5)	C29—P1—C23—C28	70.1 (5)
Ru1—N2—C8—C7	176.8 (3)	Ru1—P1—C23—C28	−166.2 (4)
C5—N2—C8—C9	178.4 (3)	C28—C23—C24—C25	−0.7 (7)
Ru1—N2—C8—C9	−2.2 (4)	P1—C23—C24—C25	176.6 (4)
C6—C7—C8—N2	0.2 (6)	C23—C24—C25—C26	−0.7 (7)
C6—C7—C8—C9	179.2 (4)	C24—C25—C26—C27	1.3 (9)
C13—N3—C9—C10	1.8 (6)	C25—C26—C27—C28	−0.5 (12)
Ru1—N3—C9—C10	179.3 (3)	C24—C23—C28—C27	1.4 (9)
C13—N3—C9—C8	−176.9 (3)	P1—C23—C28—C27	−175.9 (6)
Ru1—N3—C9—C8	0.5 (4)	C26—C27—C28—C23	−0.9 (12)
N2—C8—C9—N3	1.2 (5)	C35—P1—C29—C30	−48.5 (6)
C7—C8—C9—N3	−177.8 (3)	C23—P1—C29—C30	−154.4 (6)
N2—C8—C9—C10	−177.4 (4)	Ru1—P1—C29—C30	76.5 (6)
C7—C8—C9—C10	3.6 (6)	C35—P1—C29—C34	147.9 (5)
N3—C9—C10—C11	−0.9 (7)	C23—P1—C29—C34	42.0 (6)
C8—C9—C10—C11	177.7 (5)	Ru1—P1—C29—C34	−87.1 (6)
C9—C10—C11—C12	−0.3 (9)	C34—C29—C30—C31	−9.3 (11)
C10—C11—C12—C13	0.5 (9)	P1—C29—C30—C31	−173.3 (7)
C9—N3—C13—C12	−1.6 (6)	C29—C30—C31—C32	6.4 (15)
Ru1—N3—C13—C12	−179.0 (4)	C30—C31—C32—C33	0.2 (17)
C9—N3—C13—C14	177.5 (3)	C31—C32—C33—C34	−2.9 (18)
Ru1—N3—C13—C14	0.1 (5)	C30—C29—C34—C33	6.4 (12)
C11—C12—C13—N3	0.4 (8)	P1—C29—C34—C33	171.9 (8)
C11—C12—C13—C14	−178.6 (5)	C32—C33—C34—C29	−0.5 (17)
C18—N4—C14—C15	4.0 (6)	C23—P1—C35—C40	134.2 (5)
Ru1—N4—C14—C15	−177.8 (4)	C29—P1—C35—C40	28.2 (6)
C18—N4—C14—C13	−177.5 (3)	Ru1—P1—C35—C40	−96.7 (5)
Ru1—N4—C14—C13	0.7 (4)	C23—P1—C35—C36	−51.9 (4)
N3—C13—C14—N4	−0.6 (5)	C29—P1—C35—C36	−157.9 (4)
C12—C13—C14—N4	178.5 (5)	Ru1—P1—C35—C36	77.2 (4)
N3—C13—C14—C15	177.9 (4)	C40—C35—C36—C37	1.1 (9)
C12—C13—C14—C15	−3.0 (8)	P1—C35—C36—C37	−173.2 (5)
N4—C14—C15—C16	−0.6 (8)	C35—C36—C37—C38	−0.1 (10)
C13—C14—C15—C16	−179.1 (5)	C36—C37—C38—C39	−1.3 (11)
C14—C15—C16—C17	−2.4 (8)	C37—C38—C39—C40	1.8 (13)
C15—C16—C17—C19	−178.9 (5)	C36—C35—C40—C39	−0.6 (11)
C15—C16—C17—C18	2.0 (8)	P1—C35—C40—C39	173.4 (7)
C21—N5—C18—N4	178.1 (4)	C38—C39—C40—C35	−0.9 (14)

### cis-Aqua[2,6-bis(1,8-naphthyridin-2-yl)pyridine-κ3N,N',N''](thiocyanato-κN)(triphenylphosphine-κP)ruthenium(II) hexafluoridophosphate–acetone–water (1/0.5/1) (I) Hydrogen-bond geometry (Å, º)

**Table:**

*D*—H···*A*	*D*—H	H···*A*	*D*···*A*	*D*—H···*A*
C10—H6···F5^i^	0.93	2.45	3.369 (6)	170
C15—H9···F2	0.93	2.45	3.345 (6)	162
C21—H13···O2	0.93	2.59	3.213 (14)	124
C24—H14···O1	0.93	2.43	3.210 (5)	141
C24—H14···N5	0.93	2.43	3.144 (6)	134
C25—H15···F4^ii^	0.93	2.54	3.347 (7)	145
C41—H30···F1^ii^	0.96	2.40	3.26 (3)	150

Symmetry codes: (i) −*x*, −*y*, −*z*+2; (ii) −*x*+1, −*y*+1, −*z*+2.

### trans-[2,6-Bis(1,8-naphthyridin-2-yl)pyridine-κ3'N,N',N'']bis(pyridine-κN)(thiocyanato-κN)ruthenium(II) thiocyanate (II) Crystal data

**Table:**

[Ru(NCS)(C_21_H_13_N_5_)(C_5_H_5_N)_2_]NCS	*F*(000) = 1440.00
*M**_r_* = 710.79	*D*_x_ = 1.600 Mg m^−^^3^
Monoclinic, *P*2_1_/*c*	Mo *K*α radiation, λ = 0.71075 Å
*a* = 12.6556 (10) Å	Cell parameters from 7649 reflections
*b* = 14.0986 (7) Å	θ = 3.1–27.6°
*c* = 17.4421 (14) Å	µ = 0.72 mm^−^^1^
β = 108.535 (3)°	*T* = 93 K
*V* = 2950.7 (4) Å^3^	Platelet, purple
*Z* = 4	0.25 × 0.15 × 0.05 mm

### trans-[2,6-Bis(1,8-naphthyridin-2-yl)pyridine-κ3'N,N',N'']bis(pyridine-κN)(thiocyanato-κN)ruthenium(II) thiocyanate (II) Data collection

**Table:**

Rigaku Saturn724 diffractometer	6058 reflections with *F*^2^ > 2.0σ(*F*^2^)
Detector resolution: 28.626 pixels mm^-1^	*R*_int_ = 0.029
ω scans	θ_max_ = 27.5°, θ_min_ = 3.1°
Absorption correction: multi-scan (*REQAB*; Rigaku, 1998)	*h* = −16→16
*T*_min_ = 0.927, *T*_max_ = 0.965	*k* = −18→18
30135 measured reflections	*l* = −22→21
6758 independent reflections	

### trans-[2,6-Bis(1,8-naphthyridin-2-yl)pyridine-κ3'N,N',N'']bis(pyridine-κN)(thiocyanato-κN)ruthenium(II) thiocyanate (II) Refinement

**Table:**

Refinement on *F*^2^	Secondary atom site location: difference Fourier map
*R*[*F*^2^ > 2σ(*F*^2^)] = 0.036	Hydrogen site location: inferred from neighbouring sites
*wR*(*F*^2^) = 0.091	H-atom parameters constrained
*S* = 1.10	*w* = 1/[σ^2^(*F*_o_^2^) + (0.0451*P*)^2^ + 2.9803*P*] where *P* = (*F*_o_^2^ + 2*F*_c_^2^)/3
6758 reflections	(Δ/σ)_max_ = 0.001
406 parameters	Δρ_max_ = 1.13 e Å^−^^3^
0 restraints	Δρ_min_ = −0.81 e Å^−^^3^
Primary atom site location: structure-invariant direct methods	

### trans-[2,6-Bis(1,8-naphthyridin-2-yl)pyridine-κ3'N,N',N'']bis(pyridine-κN)(thiocyanato-κN)ruthenium(II) thiocyanate (II) Special details

**Table:**

Geometry. All esds (except the esd in the dihedral angle between two l.s. planes) are estimated using the full covariance matrix. The cell esds are taken into account individually in the estimation of esds in distances, angles and torsion angles; correlations between esds in cell parameters are only used when they are defined by crystal symmetry. An approximate (isotropic) treatment of cell esds is used for estimating esds involving l.s. planes.
Refinement. Refinement was performed using all reflections. The weighted R-factor (wR) and goodness of fit (S) are based on F^2^. R-factor (gt) are based on F. The threshold expression of F^2^ > 2.0 sigma(F^2^) is used only for calculating R-factor (gt).

### trans-[2,6-Bis(1,8-naphthyridin-2-yl)pyridine-κ3'N,N',N'']bis(pyridine-κN)(thiocyanato-κN)ruthenium(II) thiocyanate (II) Fractional atomic coordinates and isotropic or equivalent isotropic displacement parameters (Å^2^)

**Table:**

	*x*	*y*	*z*	*U*_iso_*/*U*_eq_	
Ru1	0.73332 (2)	0.38197 (2)	0.79490 (2)	0.01220 (7)	
S1	0.39862 (5)	0.39265 (5)	0.87218 (4)	0.02286 (14)	
S2	1.08750 (7)	0.12965 (7)	0.89031 (6)	0.0466 (2)	
N1	0.70294 (18)	0.38857 (15)	0.98054 (13)	0.0213 (4)	
N2	0.83872 (16)	0.38102 (13)	0.91797 (12)	0.0155 (4)	
N3	0.88229 (16)	0.37934 (13)	0.78244 (13)	0.0161 (4)	
N4	0.69378 (17)	0.37739 (13)	0.66605 (12)	0.0155 (4)	
N5	0.50056 (17)	0.38259 (15)	0.62868 (13)	0.0195 (4)	
N6	0.73064 (15)	0.52961 (14)	0.79703 (11)	0.0144 (4)	
N7	0.71689 (16)	0.23463 (14)	0.79725 (11)	0.0148 (4)	
N8	0.57797 (16)	0.38875 (13)	0.81029 (12)	0.0150 (4)	
N9	1.1118 (3)	0.1261 (2)	1.0511 (3)	0.0605 (10)	
C1	0.6735 (2)	0.38898 (19)	1.04670 (16)	0.0250 (6)	
H1	0.596645	0.395157	1.040937	0.030*	
C2	0.7502 (2)	0.38073 (18)	1.12584 (16)	0.0257 (6)	
H2	0.725046	0.381745	1.171672	0.031*	
C3	0.8603 (2)	0.37134 (19)	1.13490 (16)	0.0263 (6)	
H3	0.913218	0.365204	1.187311	0.032*	
C4	0.8955 (2)	0.37077 (18)	1.06568 (16)	0.0222 (5)	
C5	0.8121 (2)	0.37965 (16)	0.98896 (15)	0.0170 (5)	
C6	1.0075 (2)	0.3633 (2)	1.06873 (17)	0.0302 (6)	
H4	1.064516	0.355198	1.119123	0.036*	
C7	1.0338 (2)	0.3677 (2)	0.99887 (17)	0.0287 (6)	
H5	1.109374	0.363961	1.000289	0.034*	
C8	0.9484 (2)	0.37788 (17)	0.92474 (16)	0.0192 (5)	
C9	0.9741 (2)	0.38180 (16)	0.84863 (16)	0.0189 (5)	
C10	1.0804 (2)	0.38683 (18)	0.84086 (17)	0.0236 (5)	
H6	1.144589	0.388480	0.887533	0.028*	
C11	1.0912 (2)	0.38939 (18)	0.76456 (18)	0.0257 (6)	
H7	1.162986	0.394404	0.758536	0.031*	
C12	0.9974 (2)	0.38464 (18)	0.69684 (17)	0.0236 (5)	
H8	1.004151	0.385025	0.644116	0.028*	
C13	0.8932 (2)	0.37929 (16)	0.70748 (15)	0.0181 (5)	
C14	0.7866 (2)	0.37476 (16)	0.64229 (16)	0.0192 (5)	
C15	0.7840 (2)	0.3661 (2)	0.56132 (16)	0.0258 (6)	
H9	0.851351	0.365671	0.548224	0.031*	
C16	0.6846 (2)	0.3583 (2)	0.50232 (16)	0.0284 (6)	
H10	0.681492	0.349725	0.447630	0.034*	
C17	0.5856 (2)	0.36317 (19)	0.52286 (15)	0.0228 (5)	
C18	0.5922 (2)	0.37458 (16)	0.60530 (15)	0.0176 (5)	
C19	0.4790 (2)	0.3594 (2)	0.46440 (16)	0.0307 (6)	
H11	0.471574	0.350438	0.408944	0.037*	
C20	0.3871 (2)	0.3686 (2)	0.48793 (16)	0.0288 (6)	
H12	0.314452	0.367060	0.449543	0.035*	
C21	0.4027 (2)	0.38040 (18)	0.57117 (16)	0.0225 (5)	
H13	0.338013	0.387331	0.587133	0.027*	
C22	0.69873 (19)	0.57334 (17)	0.85510 (15)	0.0179 (5)	
H14	0.684341	0.535624	0.895819	0.022*	
C23	0.6862 (2)	0.67017 (18)	0.85773 (16)	0.0228 (5)	
H15	0.664358	0.698350	0.899817	0.027*	
C24	0.7058 (2)	0.72600 (18)	0.79841 (16)	0.0254 (6)	
H16	0.696320	0.792839	0.798536	0.030*	
C25	0.7396 (2)	0.68249 (18)	0.73875 (16)	0.0239 (5)	
H17	0.754158	0.719108	0.697497	0.029*	
C26	0.7518 (2)	0.58469 (18)	0.74030 (15)	0.0189 (5)	
H18	0.775997	0.555269	0.699798	0.023*	
C27	0.8032 (2)	0.17690 (17)	0.83344 (14)	0.0174 (5)	
H19	0.875293	0.203965	0.855670	0.021*	
C28	0.7914 (2)	0.07944 (18)	0.83963 (15)	0.0206 (5)	
H20	0.854209	0.040994	0.865668	0.025*	
C29	0.6872 (2)	0.03930 (18)	0.80747 (16)	0.0236 (5)	
H21	0.677155	−0.027208	0.810502	0.028*	
C30	0.5974 (2)	0.09807 (18)	0.77057 (16)	0.0215 (5)	
H22	0.524601	0.072233	0.748726	0.026*	
C31	0.6145 (2)	0.19431 (17)	0.76586 (15)	0.0183 (5)	
H23	0.552640	0.233747	0.739781	0.022*	
C32	0.50337 (19)	0.38985 (16)	0.83534 (14)	0.0156 (4)	
C33	1.1013 (3)	0.1275 (2)	0.9913 (3)	0.0385 (8)	

### trans-[2,6-Bis(1,8-naphthyridin-2-yl)pyridine-κ3'N,N',N'']bis(pyridine-κN)(thiocyanato-κN)ruthenium(II) thiocyanate (II) Atomic displacement parameters (Å^2^)

**Table:**

	*U*^11^	*U*^22^	*U*^33^	*U*^12^	*U*^13^	*U*^23^
Ru1	0.01117 (10)	0.01297 (10)	0.01255 (10)	0.00016 (6)	0.00390 (7)	0.00024 (7)
S1	0.0170 (3)	0.0297 (3)	0.0251 (3)	−0.0005 (2)	0.0111 (2)	−0.0020 (3)
S2	0.0280 (4)	0.0616 (6)	0.0502 (5)	0.0079 (4)	0.0122 (4)	0.0023 (4)
N1	0.0198 (10)	0.0278 (11)	0.0160 (10)	0.0031 (8)	0.0055 (8)	0.0019 (8)
N2	0.0145 (9)	0.0156 (9)	0.0144 (10)	−0.0001 (7)	0.0020 (8)	0.0011 (7)
N3	0.0155 (9)	0.0147 (9)	0.0196 (10)	−0.0002 (7)	0.0076 (8)	0.0009 (8)
N4	0.0182 (9)	0.0157 (9)	0.0139 (9)	0.0003 (7)	0.0069 (8)	0.0010 (7)
N5	0.0183 (10)	0.0246 (11)	0.0153 (10)	0.0000 (8)	0.0049 (8)	−0.0010 (8)
N6	0.0131 (9)	0.0137 (9)	0.0141 (9)	0.0001 (7)	0.0012 (7)	0.0013 (7)
N7	0.0168 (9)	0.0139 (9)	0.0149 (9)	−0.0010 (7)	0.0069 (8)	−0.0012 (7)
N8	0.0143 (9)	0.0163 (9)	0.0138 (9)	0.0001 (7)	0.0036 (7)	−0.0001 (7)
N9	0.074 (2)	0.0450 (19)	0.092 (3)	−0.0064 (16)	0.068 (2)	−0.0125 (19)
C1	0.0267 (13)	0.0310 (14)	0.0201 (13)	0.0053 (11)	0.0112 (11)	0.0029 (10)
C2	0.0354 (15)	0.0266 (14)	0.0170 (12)	0.0003 (11)	0.0107 (11)	0.0011 (10)
C3	0.0320 (14)	0.0269 (14)	0.0150 (12)	−0.0016 (11)	0.0005 (11)	0.0023 (10)
C4	0.0236 (12)	0.0209 (12)	0.0176 (12)	−0.0002 (10)	0.0003 (10)	0.0013 (10)
C5	0.0189 (11)	0.0148 (11)	0.0163 (11)	0.0001 (9)	0.0042 (9)	0.0007 (9)
C6	0.0203 (13)	0.0416 (16)	0.0218 (13)	−0.0003 (11)	−0.0031 (11)	0.0040 (12)
C7	0.0169 (12)	0.0390 (16)	0.0260 (14)	0.0010 (11)	0.0009 (11)	0.0041 (12)
C8	0.0144 (11)	0.0178 (11)	0.0236 (13)	−0.0003 (9)	0.0036 (10)	0.0015 (9)
C9	0.0152 (11)	0.0172 (11)	0.0236 (13)	0.0000 (9)	0.0054 (10)	0.0011 (9)
C10	0.0158 (11)	0.0237 (13)	0.0310 (14)	0.0001 (9)	0.0070 (10)	0.0003 (11)
C11	0.0168 (12)	0.0256 (13)	0.0387 (16)	−0.0012 (10)	0.0145 (11)	0.0020 (11)
C12	0.0221 (12)	0.0245 (13)	0.0297 (14)	0.0003 (10)	0.0163 (11)	0.0012 (11)
C13	0.0197 (12)	0.0174 (11)	0.0202 (12)	0.0012 (9)	0.0105 (10)	0.0010 (9)
C14	0.0197 (12)	0.0167 (11)	0.0231 (13)	0.0015 (9)	0.0094 (10)	0.0000 (9)
C15	0.0268 (13)	0.0329 (15)	0.0223 (13)	0.0019 (11)	0.0142 (11)	0.0023 (11)
C16	0.0318 (14)	0.0399 (16)	0.0173 (13)	0.0023 (12)	0.0128 (11)	0.0005 (11)
C17	0.0263 (13)	0.0281 (13)	0.0145 (12)	0.0015 (10)	0.0073 (10)	0.0023 (10)
C18	0.0200 (11)	0.0161 (11)	0.0168 (12)	−0.0008 (9)	0.0058 (9)	−0.0011 (9)
C19	0.0356 (15)	0.0422 (16)	0.0135 (12)	0.0007 (13)	0.0068 (11)	−0.0013 (11)
C20	0.0231 (13)	0.0411 (16)	0.0178 (13)	−0.0005 (11)	0.0001 (10)	−0.0006 (11)
C21	0.0206 (12)	0.0281 (14)	0.0183 (12)	−0.0006 (10)	0.0053 (10)	−0.0018 (10)
C22	0.0174 (11)	0.0193 (12)	0.0165 (11)	0.0009 (9)	0.0045 (9)	0.0009 (9)
C23	0.0239 (12)	0.0205 (12)	0.0221 (13)	0.0037 (10)	0.0048 (10)	−0.0024 (10)
C24	0.0323 (14)	0.0141 (12)	0.0264 (14)	0.0032 (10)	0.0045 (11)	0.0009 (10)
C25	0.0291 (13)	0.0181 (12)	0.0223 (13)	−0.0018 (10)	0.0049 (11)	0.0036 (10)
C26	0.0192 (11)	0.0188 (11)	0.0172 (12)	−0.0010 (9)	0.0038 (9)	0.0023 (9)
C27	0.0170 (11)	0.0196 (12)	0.0159 (11)	0.0016 (9)	0.0057 (9)	−0.0010 (9)
C28	0.0248 (12)	0.0186 (12)	0.0199 (12)	0.0046 (10)	0.0092 (10)	0.0021 (10)
C29	0.0362 (14)	0.0155 (12)	0.0225 (13)	−0.0025 (10)	0.0144 (11)	−0.0007 (10)
C30	0.0225 (12)	0.0209 (12)	0.0214 (13)	−0.0065 (10)	0.0075 (10)	−0.0044 (10)
C31	0.0172 (11)	0.0194 (12)	0.0178 (12)	−0.0013 (9)	0.0050 (9)	−0.0031 (9)
C32	0.0163 (11)	0.0148 (11)	0.0133 (11)	0.0006 (8)	0.0015 (9)	0.0001 (8)
C33	0.0233 (14)	0.0296 (16)	0.056 (2)	−0.0018 (12)	0.0027 (15)	0.0152 (15)

### trans-[2,6-Bis(1,8-naphthyridin-2-yl)pyridine-κ3'N,N',N'']bis(pyridine-κN)(thiocyanato-κN)ruthenium(II) thiocyanate (II) Geometric parameters (Å, º)

**Table:**

Ru1—N3	1.966 (2)	C10—C11	1.381 (4)
Ru1—N8	2.069 (2)	C10—H6	0.9500
Ru1—N6	2.0824 (19)	C11—C12	1.384 (4)
Ru1—N7	2.0893 (19)	C11—H7	0.9500
Ru1—N2	2.137 (2)	C12—C13	1.391 (3)
Ru1—N4	2.142 (2)	C12—H8	0.9500
S1—C32	1.647 (2)	C13—C14	1.464 (4)
S2—C33	1.715 (4)	C14—C15	1.407 (4)
N1—C1	1.320 (3)	C15—C16	1.353 (4)
N1—C5	1.348 (3)	C15—H9	0.9500
N2—C8	1.355 (3)	C16—C17	1.410 (4)
N2—C5	1.383 (3)	C16—H10	0.9500
N3—C9	1.353 (3)	C17—C19	1.410 (4)
N3—C13	1.358 (3)	C17—C18	1.423 (3)
N4—C14	1.365 (3)	C19—C20	1.357 (4)
N4—C18	1.382 (3)	C19—H11	0.9500
N5—C21	1.323 (3)	C20—C21	1.412 (4)
N5—C18	1.351 (3)	C20—H12	0.9500
N6—C26	1.350 (3)	C21—H13	0.9500
N6—C22	1.352 (3)	C22—C23	1.377 (4)
N7—C27	1.348 (3)	C22—H14	0.9500
N7—C31	1.361 (3)	C23—C24	1.384 (4)
N8—C32	1.160 (3)	C23—H15	0.9500
N9—C33	1.009 (5)	C24—C25	1.387 (4)
C1—C2	1.417 (4)	C24—H16	0.9500
C1—H1	0.9500	C25—C26	1.387 (4)
C2—C3	1.358 (4)	C25—H17	0.9500
C2—H2	0.9500	C26—H18	0.9500
C3—C4	1.413 (4)	C27—C28	1.390 (3)
C3—H3	0.9500	C27—H19	0.9500
C4—C6	1.407 (4)	C28—C29	1.379 (4)
C4—C5	1.422 (3)	C28—H20	0.9500
C6—C7	1.363 (4)	C29—C30	1.389 (4)
C6—H4	0.9500	C29—H21	0.9500
C7—C8	1.405 (4)	C30—C31	1.381 (3)
C7—H5	0.9500	C30—H22	0.9500
C8—C9	1.465 (4)	C31—H23	0.9500
C9—C10	1.396 (3)		
			
N3—Ru1—N8	178.11 (8)	C10—C11—H7	120.0
N3—Ru1—N6	92.43 (7)	C12—C11—H7	120.0
N8—Ru1—N6	85.99 (7)	C11—C12—C13	118.7 (2)
N3—Ru1—N7	95.07 (7)	C11—C12—H8	120.6
N8—Ru1—N7	86.49 (7)	C13—C12—H8	120.6
N6—Ru1—N7	172.39 (7)	N3—C13—C12	121.3 (2)
N3—Ru1—N2	78.26 (8)	N3—C13—C14	113.4 (2)
N8—Ru1—N2	100.68 (8)	C12—C13—C14	125.3 (2)
N6—Ru1—N2	89.82 (7)	N4—C14—C15	124.0 (2)
N7—Ru1—N2	90.47 (7)	N4—C14—C13	115.6 (2)
N3—Ru1—N4	78.22 (8)	C15—C14—C13	120.3 (2)
N8—Ru1—N4	102.88 (8)	C16—C15—C14	119.3 (2)
N6—Ru1—N4	92.82 (7)	C16—C15—H9	120.3
N7—Ru1—N4	89.95 (7)	C14—C15—H9	120.3
N2—Ru1—N4	156.42 (8)	C15—C16—C17	119.3 (2)
C1—N1—C5	118.0 (2)	C15—C16—H10	120.4
C8—N2—C5	117.1 (2)	C17—C16—H10	120.4
C8—N2—Ru1	112.50 (16)	C16—C17—C19	122.5 (2)
C5—N2—Ru1	130.37 (16)	C16—C17—C18	119.4 (2)
C9—N3—C13	119.9 (2)	C19—C17—C18	118.1 (2)
C9—N3—Ru1	119.92 (17)	N5—C18—N4	116.4 (2)
C13—N3—Ru1	120.09 (16)	N5—C18—C17	122.3 (2)
C14—N4—C18	116.5 (2)	N4—C18—C17	121.3 (2)
C14—N4—Ru1	112.51 (16)	C20—C19—C17	119.5 (2)
C18—N4—Ru1	130.93 (16)	C20—C19—H11	120.2
C21—N5—C18	117.1 (2)	C17—C19—H11	120.2
C26—N6—C22	117.6 (2)	C19—C20—C21	118.0 (3)
C26—N6—Ru1	123.63 (16)	C19—C20—H12	121.0
C22—N6—Ru1	118.65 (16)	C21—C20—H12	121.0
C27—N7—C31	117.6 (2)	N5—C21—C20	125.0 (2)
C27—N7—Ru1	122.59 (16)	N5—C21—H13	117.5
C31—N7—Ru1	119.71 (16)	C20—C21—H13	117.5
C32—N8—Ru1	166.03 (19)	N6—C22—C23	122.8 (2)
N1—C1—C2	123.7 (3)	N6—C22—H14	118.6
N1—C1—H1	118.1	C23—C22—H14	118.6
C2—C1—H1	118.1	C22—C23—C24	119.3 (2)
C3—C2—C1	118.7 (3)	C22—C23—H15	120.3
C3—C2—H2	120.6	C24—C23—H15	120.3
C1—C2—H2	120.6	C23—C24—C25	118.7 (2)
C2—C3—C4	119.4 (2)	C23—C24—H16	120.6
C2—C3—H3	120.3	C25—C24—H16	120.6
C4—C3—H3	120.3	C26—C25—C24	118.9 (2)
C6—C4—C3	123.8 (2)	C26—C25—H17	120.5
C6—C4—C5	118.7 (2)	C24—C25—H17	120.5
C3—C4—C5	117.6 (2)	N6—C26—C25	122.6 (2)
N1—C5—N2	115.7 (2)	N6—C26—H18	118.7
N1—C5—C4	122.6 (2)	C25—C26—H18	118.7
N2—C5—C4	121.7 (2)	N7—C27—C28	122.8 (2)
C7—C6—C4	119.5 (2)	N7—C27—H19	118.6
C7—C6—H4	120.2	C28—C27—H19	118.6
C4—C6—H4	120.2	C29—C28—C27	119.1 (2)
C6—C7—C8	119.5 (2)	C29—C28—H20	120.4
C6—C7—H5	120.3	C27—C28—H20	120.4
C8—C7—H5	120.3	C28—C29—C30	118.7 (2)
N2—C8—C7	123.4 (2)	C28—C29—H21	120.7
N2—C8—C9	115.9 (2)	C30—C29—H21	120.7
C7—C8—C9	120.7 (2)	C31—C30—C29	119.5 (2)
N3—C9—C10	120.7 (2)	C31—C30—H22	120.2
N3—C9—C8	113.2 (2)	C29—C30—H22	120.2
C10—C9—C8	126.1 (2)	N7—C31—C30	122.3 (2)
C11—C10—C9	119.2 (2)	N7—C31—H23	118.9
C11—C10—H6	120.4	C30—C31—H23	118.9
C9—C10—H6	120.4	N8—C32—S1	179.0 (2)
C10—C11—C12	120.1 (2)	N9—C33—S2	178.4 (4)
			
C5—N1—C1—C2	0.1 (4)	Ru1—N4—C14—C15	176.3 (2)
N1—C1—C2—C3	−0.4 (4)	C18—N4—C14—C13	179.1 (2)
C1—C2—C3—C4	0.5 (4)	Ru1—N4—C14—C13	−2.3 (2)
C2—C3—C4—C6	178.7 (3)	N3—C13—C14—N4	4.0 (3)
C2—C3—C4—C5	−0.4 (4)	C12—C13—C14—N4	−175.0 (2)
C1—N1—C5—N2	−179.1 (2)	N3—C13—C14—C15	−174.6 (2)
C1—N1—C5—C4	0.0 (4)	C12—C13—C14—C15	6.4 (4)
C8—N2—C5—N1	176.2 (2)	N4—C14—C15—C16	−1.0 (4)
Ru1—N2—C5—N1	−6.5 (3)	C13—C14—C15—C16	177.5 (2)
C8—N2—C5—C4	−2.9 (3)	C14—C15—C16—C17	2.7 (4)
Ru1—N2—C5—C4	174.35 (17)	C15—C16—C17—C19	177.5 (3)
C6—C4—C5—N1	−179.0 (2)	C15—C16—C17—C18	−1.0 (4)
C3—C4—C5—N1	0.1 (4)	C21—N5—C18—N4	179.7 (2)
C6—C4—C5—N2	0.0 (4)	C21—N5—C18—C17	−0.7 (3)
C3—C4—C5—N2	179.2 (2)	C14—N4—C18—N5	−176.4 (2)
C3—C4—C6—C7	−177.0 (3)	Ru1—N4—C18—N5	5.3 (3)
C5—C4—C6—C7	2.1 (4)	C14—N4—C18—C17	4.0 (3)
C4—C6—C7—C8	−1.2 (4)	Ru1—N4—C18—C17	−174.26 (18)
C5—N2—C8—C7	3.9 (3)	C16—C17—C18—N5	177.9 (2)
Ru1—N2—C8—C7	−173.8 (2)	C19—C17—C18—N5	−0.6 (4)
C5—N2—C8—C9	−178.5 (2)	C16—C17—C18—N4	−2.5 (4)
Ru1—N2—C8—C9	3.7 (2)	C19—C17—C18—N4	179.0 (2)
C6—C7—C8—N2	−1.9 (4)	C16—C17—C19—C20	−177.1 (3)
C6—C7—C8—C9	−179.3 (3)	C18—C17—C19—C20	1.3 (4)
C13—N3—C9—C10	1.7 (3)	C17—C19—C20—C21	−0.8 (4)
Ru1—N3—C9—C10	−175.39 (18)	C18—N5—C21—C20	1.3 (4)
C13—N3—C9—C8	−177.8 (2)	C19—C20—C21—N5	−0.5 (4)
Ru1—N3—C9—C8	5.1 (3)	C26—N6—C22—C23	0.7 (3)
N2—C8—C9—N3	−5.7 (3)	Ru1—N6—C22—C23	−174.93 (18)
C7—C8—C9—N3	171.9 (2)	N6—C22—C23—C24	0.6 (4)
N2—C8—C9—C10	174.8 (2)	C22—C23—C24—C25	−1.2 (4)
C7—C8—C9—C10	−7.5 (4)	C23—C24—C25—C26	0.4 (4)
N3—C9—C10—C11	0.1 (4)	C22—N6—C26—C25	−1.5 (3)
C8—C9—C10—C11	179.5 (2)	Ru1—N6—C26—C25	173.92 (18)
C9—C10—C11—C12	−1.6 (4)	C24—C25—C26—N6	0.9 (4)
C10—C11—C12—C13	1.3 (4)	C31—N7—C27—C28	0.0 (3)
C9—N3—C13—C12	−2.0 (3)	Ru1—N7—C27—C28	175.82 (17)
Ru1—N3—C13—C12	175.11 (18)	N7—C27—C28—C29	0.1 (4)
C9—N3—C13—C14	179.0 (2)	C27—C28—C29—C30	−0.6 (4)
Ru1—N3—C13—C14	−3.9 (3)	C28—C29—C30—C31	1.0 (4)
C11—C12—C13—N3	0.5 (4)	C27—N7—C31—C30	0.4 (3)
C11—C12—C13—C14	179.4 (2)	Ru1—N7—C31—C30	−175.53 (18)
C18—N4—C14—C15	−2.3 (3)	C29—C30—C31—N7	−0.9 (4)

### trans-[2,6-Bis(1,8-naphthyridin-2-yl)pyridine-κ3'N,N',N'']bis(pyridine-κN)(thiocyanato-κN)ruthenium(II) thiocyanate (II) Hydrogen-bond geometry (Å, º)

**Table:**

*D*—H···*A*	*D*—H	H···*A*	*D*···*A*	*D*—H···*A*
C12—H8···N9^i^	0.95	2.43	3.305 (5)	152
C20—H12···S2^ii^	0.95	2.73	3.629 (3)	159
C22—H14···N1	0.95	2.51	3.391 (3)	154
C27—H19···S2	0.95	2.76	3.479 (3)	133

Symmetry codes: (i) *x*, −*y*+1/2, *z*−1/2; (ii) *x*−1, −*y*+1/2, *z*−1/2.
